# HRAS mutation as a diagnostic molecular analysis for epithelial‐myoepithelial carcinoma of the parotid gland: A case report

**DOI:** 10.1002/ccr3.8099

**Published:** 2023-10-23

**Authors:** Taisei Yasuda, Masami Osaki, Masahiko Sugitani

**Affiliations:** ^1^ Department of Otolaryngology‐Head and Neck Surgery Ageo Central General Hospital Saitama Japan; ^2^ Department of Pathology Ageo Central General Hospital Saitama Japan

**Keywords:** epithelial‐myoepithelial carcinoma, *HRAS* mutation, salivary gland carcinoma

## Abstract

*HRAS* mutations are frequent genetic alterations in epithelial‐myoepithelial carcinoma, and they may be useful as ancillary molecular tests and predictive molecular tests for targeted therapy with tipifarnib.

## Graphical abstract

Epithelial‐myoepithelial carcinoma has a biphasic appearance with ductal epithelial and myoepithelial cells and a wide spectrum of histologic presentations. Because of its histologic variety, it is sometimes challenging to make an accurate diagnosis.

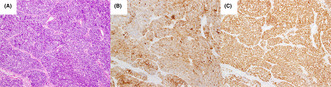

## 1 INTRODUCTION

Epithelial‐myoepithelial carcinoma (EMC) is a rare salivary gland neoplasm that accounts for less than 1% of the incidence of all salivary gland tumors.^1^ EMC has a biphasic appearance with ductal epithelial and myoepithelial cells and a wide spectrum of histologic presentations. Therefore, it is often difficult to diagnose. *HRAS* mutations are frequent genetic alterations in EMC,^2^ although there are only a few published reports on their detection for diagnostic purposes. Herein, we describe two cases in which the detection of an *HRAS* mutation supported the diagnosis of EMC.

## 2 CASE PRESENTATION

### 2.1 Patient 1

**FIGURE 1:**
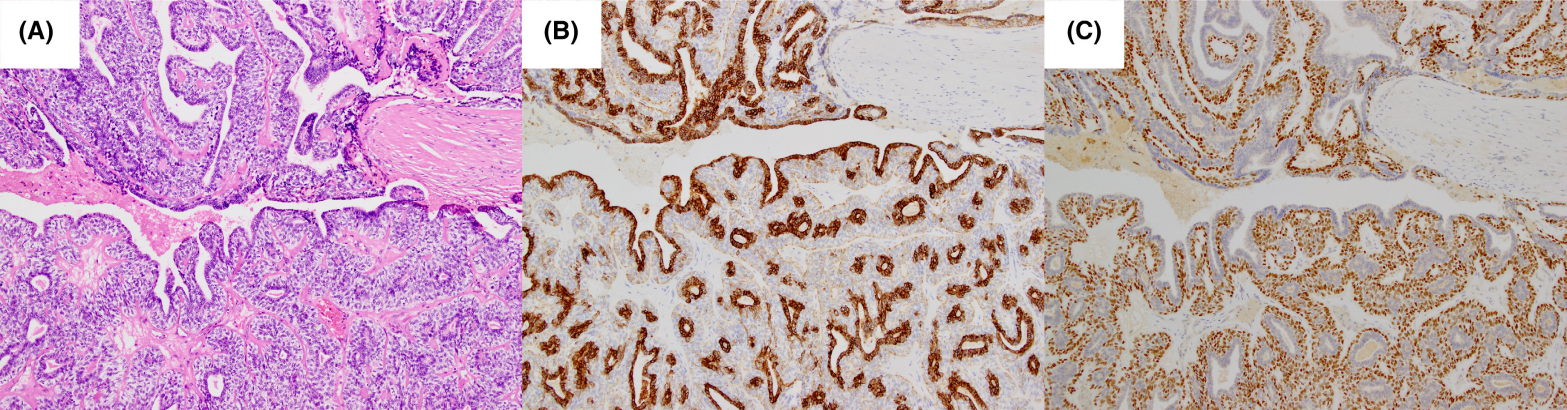
(A) Carcinoma cells are observed as biphasic, ductal, and myoepithelial cells (H&E staining; 100× magnification). (B) Luminal cells are positive for cytokeratin (cytokeratin AE1/AE3 immunostaining; 100× magnification) with (C) strong nuclear p63 staining (p63 immunohistochemistry; 100× magnification).

An 83‐year‐old woman presented with painless swelling in her right parotid gland without facial paralysis. Neck contrast‐enhanced computed tomography showed a primary parotid tumor (24 × 19 × 16 mm in size) and no cervical metastases. Fluorine‐18 fluoro‐2‐deoxy‐D‐glucose (FDG)‐positron emission tomography showed abnormally high FDG uptake in the right parotid gland. Superior parotidectomy was performed without capsule rupture and with free margins; the pathologic stage was pT2N0M0. Pathologically, the tumor was multinodular with biphasic tubules and solid nests (Figure 1A). Immunostaining showed positivity for cytokeratin (CK) AE1/AE3 in ductal epithelial cells and smooth muscle actin and p63 in myoepithelial‐like cells (Figure 1B, C). The Ki‐67 labeling index (LI) was approximately 12%. Sanger sequencing revealed the *HRAS* Q61R mutation. Thus, the patient was diagnosed with EMC. 1‐year follow‐up revealed no local recurrence or metastasis.

### 2.2 Patient 2

**FIGURE 2:**
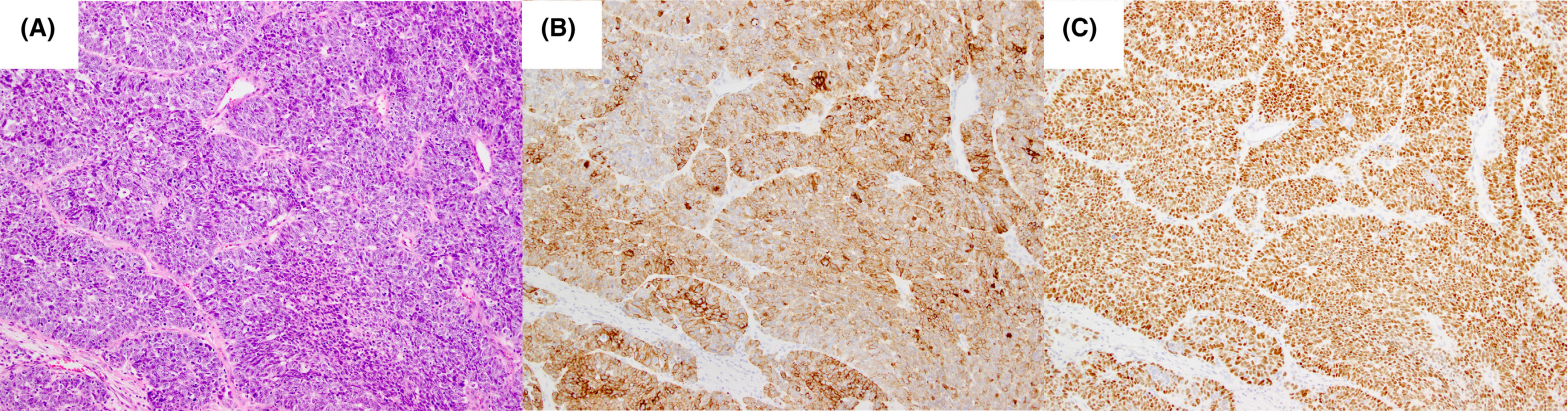
(A) Epithelial cell proliferation with partial glandular duct formation (H&E staining; 10× magnification). (B) Luminal cells are positive for cytokeratin (cytokeratin AE1/AE3 immunostaining; 10× magnification) with (C) intense staining for p63 (p63 immunohistochemistry; 10× magnification).

A 64‐year‐old man was referred for right parotid swelling with facial palsy (75/100: Sunnybrook method). Magnetic resonance imaging showed a mass (45 × 30 × 26 mm in size) in the right parotid gland with suspected infiltration of the surrounding soft tissue and mandible. Parotid carcinoma stage cT4aN0M0 was diagnosed, and extended parotidectomy, ipsilateral selective neck dissection, facial nerve reconstruction, and anterolateral thigh flap reconstruction were performed. Histopathology of the resected tumor showed a salivary gland carcinoma with biphasic appearance composed mainly of basaloid cells with marked atypia (Figure 2A). Immunostaining showed positivity for CK AE1/AE3, CK7, p40, DOG1, and p63 (Figure 2B,C). S‐100 protein, α‐smooth muscle actin, vimentin, androgen receptor, human epidermal growth factor receptor 2, CD56, chromogranin, synaptophysin, and β‐catenin were negatively stained. The Ki‐67 LI was approximately 70% in neoplastic cells. The differential diagnosis included basal cell adenoma (BCA)/basal cell adenocarcinoma (BCAC) and adenoid cystic carcinoma (ACC). Sanger sequencing revealed the *HRAS* Q61K mutation (no mutation in *HRAS* codons 12 and 13). Thus, the final diagnosis was EMC. Since histopathology showed a high‐grade carcinoma, we suspected EMC with high‐grade transformation. The postoperative course was uneventful. The patient was disease‐free at 24 months.

## 3 DISCUSSION

EMC has several histologic variations, and it is difficult to accurately diagnose.^2^ Immunohistochemical staining is of limited value in differentiating EMC from other salivary gland tumors with biphasic differentiation. However, *HRAS* mutation testing may increase the rate of accurate diagnosis.

Molecular analysis has become recognized globally for its importance in the diagnosis of salivary gland cancers. Therefore, salivary gland tumors are increasingly being subjected to molecular testing for differential diagnosis and suitable therapeutic therapy.^3^ *HRAS* mutations are present in 81.7% of EMCs. However, no *HRAS* mutations have been identified in EMC‐like salivary gland tumors, such as ACC, pleomorphic adenoma, BCA/BCAC, and myoepithelial carcinoma.^2^ Herein, both cases lacked characteristic features of EMC, although the presence of *HRAS* mutations led to a diagnosis. Additionally, we distinguished EMC from its histologic mimic more accurately by confirming the absence of β‐catenin nuclear reactivity observed in BCA/BCAC and non‐detection of *MYB* mutations by fluorescence in situ hybridization, indicating ACC. Notably, there is no significant correlation between the *HRAS* mutation status and histologic indicators of tumor aggressiveness.^2^ Furthermore, the presence of *HRAS* mutations alone is not sufficient to diagnose EMC because *they* have also been observed in other subtypes of salivary gland cancers, including salivary duct carcinomas.

EMC is generally low‐grade. However, high‐grade transformations (HGTs) have been reported.^1^, ^4^ Dedifferentiation of salivary gland tumors has been described as an HGT, where a low‐grade carcinoma results in a secondary high‐grade carcinoma. This is associated with a worse prognosis. For Case 2, a high degree of necrosis, numerous mitoses, and a high Ki‐67 LI were observed, indicating a high‐grade tumor. It was difficult to conclude that it was an HGT of EMC based on the histopathological features. However, the *HRAS* mutation led to the diagnosis of EMC.

The standard treatment for patients with localized EMC is complete surgical resection. The prognosis is relatively good for patients who undergo wide surgical resection with clear margins.^5^ To the best of our knowledge, there are no standard therapy options for those with recurrent, incurable, or metastatic disease. *HRAS* mutations may serve as diagnostic and predictive molecular testing for targeted therapy with tipifarnib for recurrent or metastatic cases.^6^

## 4 CONCLUSION

Genetic analysis of *HRAS* mutations is a crucial adjunct to the pathological diagnosis of EMC and may play a decisive role, especially for difficult‐to‐diagnose cases.

## AUTHOR CONTRIBUTIONS

**Taisei Yasuda:** Conceptualization; data curation; writing – original draft. **Masami Osaki:** Data curation; formal analysis; supervision; validation. **Masahiko Sugitani:** Data curation; formal analysis; supervision; validation; visualization.

## FUNDING INFORMATION

This research received no specific grant from any funding agency in the public, commercial, or not‐for‐profit sectors.

## CONFLICT OF INTEREST STATEMENT

The authors have no conflicts of interest to declare.

## ETHICS STATEMENT

This study protocol was reviewed and approved by the Ageo Central General Hospital Institutional Review Board.

## CONSENT

Written informed consent was obtained from the patient to publish this report according to the patient consent policy of the journal.

## Data Availability

The datasets generated during and/or analyzed during the current study are available from the corresponding author upon reasonable request.
